# QuickStats

**Published:** 2013-01-18

**Authors:** Bruce A. Dye, Xianfen Li

**Figure:**
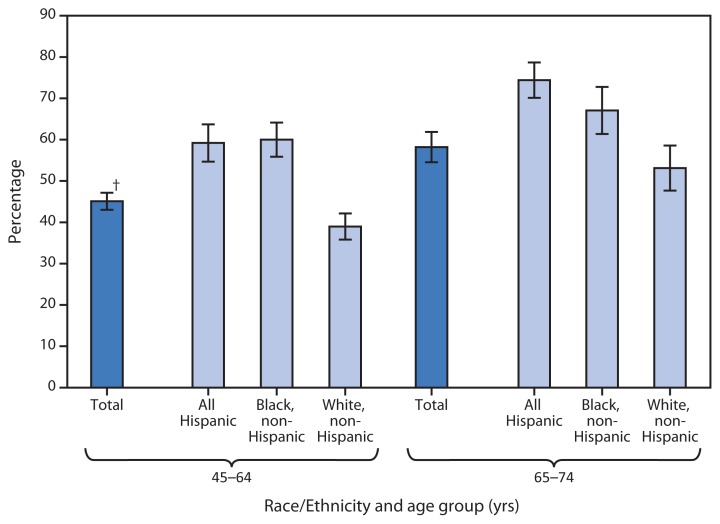
Prevalence of Moderate and Severe Periodontitis* Among Adults Aged 45–74 Years, by Race/Ethnicity and Age Group — National Health and Nutrition Examination Survey, United States, 2009–2010 * Severe periodontitis was defined as the presence of two or more interproximal sites with ≥6 mm attachment loss and one or more interproximal sites with ≥5 mm probing depth. Moderate periodontitis was defined as two or more interproximal sites with ≥4 mm attachment loss or two or more interproximal sites with ≥5 mm probing depth. ^†^ 95% confidence interval.

During 2009–2010, 45% of adults aged 45–64 years had moderate or severe periodontitis. In that age group, the prevalence of moderate or severe periodontitis was significantly higher for Hispanic and non-Hispanic black adults (59% and 60%, respectively) compared with non-Hispanic white adults (39%). Among adults aged 65–74 years, 58% had moderate or severe periodontitis. Hispanics had a higher prevalence of periodontitis (74%) compared with non-Hispanic whites (53%).

**Source:** Eke PI, Dye BA, Wei L, Thornton-Evans GO, Genco RJ. Prevalence of periodontitis in adults in the United States: 2009 and 2010. J Dent Res 2012;91:914–20.

